# Low-Temperature Superplasticity and High Strength in the Al 2024 Alloy with Ultrafine Grains

**DOI:** 10.3390/ma16020727

**Published:** 2023-01-11

**Authors:** Elena V. Bobruk, Maxim Yu. Murashkin, Ilnar A. Ramazanov, Vil U. Kazykhanov, Ruslan Z. Valiev

**Affiliations:** 1 Institute of Physics of Advanced Materials, Ufa University of Science and Technology, 32 Zaki Validi Str., Ufa 450076, Russia; 2 Laboratory of Multifunctional Materials, “Higher Engineering School for Aerospace Technologies” Center, Ufa University of Science and Technology, 32 Zaki Validi Str., Ufa 450076, Russia

**Keywords:** superplasticity, aluminum alloys, ultrafine-grained materials, strength

## Abstract

This study aims to achieve an ultrafine-grained (UFG) Al 2024 alloy superplasticity at temperatures lower than the traditional ones for commercial Al alloys (400–500 °C). The UFG structure with a mean grain size of 100 nm produced in the alloy by high-pressure torsion at room temperature provided a very high strength—microhardness (HV_0.1_) of 286 ± 4, offset yield strength (σ_0.2_) of 828 ± 9 MPa, and ultimate tensile strength (σ_UTS_) of 871 ± 6 MPa at elongation to failure (δ) of 7 ± 0.2%. Complex tensile tests were performed at temperatures from 190 to 270 °C and strain rates from 10^−2^ to 5 × 10^−5^ s^−1^, and the values of flow stress, total elongation and strain rate-sensitivity coefficient were determined. The UFG alloy was shown to exhibit superplastic behavior at test temperatures of 240 and 270 °C. For the first time, 400% elongation was achieved in the alloy at an unusually low temperature of 270 °C (0.56 T_m_) and strain rate of 10^−3^ s^−1^. The UFG 2024 alloy after superplastic deformation was found to have higher strength (150–160 HV) than that after the standard strengthening heat treatment T6.

## 1. Introduction

The Al-based materials for structural applications that are in most demand include the 2xxx series Al-Cu-Mg alloys [1,2,3]. At present, they are widely used in the aerospace industry and transport. Alloys of this system are age-hardened materials that are hardened by heat treatment or thermomechanical treatment (TMT). Alongside Al-Mg and Al-Zn-Mg-Cu alloys, Al-Cu-Mg alloys exhibit superior strength properties in the ultrafine-grained (UFG) states produced by severe plastic deformation (SPD) techniques [4,5,6]. It was shown that the UFG structure formation in the D16 alloy (analogue of the 2024 alloy) by high-pressure torsion (HPT) at room temperature enabled a UTS of over 1000 MPa to be achieved [5]. Such a high level of properties produced in the material under study is determined, in addition to grain refinement to 70 nm and a high dislocation density, by an interaction of dislocations with the precipitates of the particles of the T(Al_20_Cu_2_Mn_3_) and S(Al_2_CuMg) phases which control the intensity of dynamic recovery and recrystallization. However, the high strength of this UFG Al alloy 2024 is quickly lost after heating above 200 °C when recrystallization processes occur in the alloy and grain growth takes place [7,8]. In this connection, the superplastic forming of this alloy realized at temperatures of 450–500 °C for producing complex-shaped items ensures, in these items, strength below 250–300 MPa. Therefore, after such forming an additional heat treatment of the T6 type is traditionally introduced, which raises strength to 450 MPa due to aging, but this makes the process much more expensive [9,10].

In this regard, in recent years a lot of attention has been paid to the studies of the superplasticity of Al alloys with a UFG structure in the submicrometer and nanometer ranges [6,11,12], which enables their temperature of superplasticity to be considerably decreased. For instance, in the UFG Al 7075 alloy (Al-Zn-Mg-Zr system) produced by HPT, the temperature of superplasticity was decreased below 200 °C, which enabled a small grain size to be preserved and provided a very high strength even after superplastic deformation [12].

In this work, we studied for the first time the superplasticity of the 2024 alloy with a UFG structure produced by HPT. The aim of this research was to study the superplastic behavior of this alloy at lower temperatures and the effect of this processing on its strength properties at room temperature. The revealed features of the mechanical behavior of Al alloys with UFG structures may create new opportunities for the design of advanced lightweight and high-strength products and structures for the application in both the automotive and aerospace industries.

## 2. Materials and Methods

The conventionally extruded Al alloy 2024 with a chemical composition presented in Table 1, in weight percent, was cut into rods with a diameter of 20 mm and length of 150 mm. The chemical composition of the alloy was qualitatively analyzed using a Bruker Q4 Tasman optical emission spectrometer. 

The initial billets of the studied alloy were in the hot-extruded state with a coarse-grained structure that typically forms in the semi-finished products after pressing. The width of the fibers was about 30 µm, the length was 65 µm. The 2024 alloy in the initial (as-received) state had a low microhardness with a mean value of HV_0.1_ = 86 ± 4. The initial alloy samples were annealed at a temperature of 497 ± 5 °C for 2 h before straining and then quenched in water for solid-solution hardening, the maximum refinement of the grain structure during severe plastic deformation (SPD) and maintaining the potential for further hardening of the studied material due to precipitation hardening and/or dislocation hardening. It is after quenching that the initial state in thermally hardened aluminum alloys is rational for their subsequent SPD processing to form a UFG structure and achieve the best combination of strength and ductility characteristics. 

For the enhancement of strength properties, Al-Cu-Mg alloys are subjected to quenching with subsequent natural or artificial aging [1,2,3,10]. Therefore, to compare the results after quenching, some of the samples were subjected to artificial aging at a temperature of 190 °C for 10 h [10]. This conventional treatment regime corresponds to the T6 hardening treatment for maximum strength. The billets were processed by SPD no more than 1.5 h after the heat treatment to preserve the maximum effect of quenching.

The samples of the required geometry were prepared on a precision 2-coordinate wire-cutting electroerosive machine of the ARTA-120 model (OOO “NPK Delta-Test”, Fryazino, Russia) in water at room temperature (to eliminate the effect of thermal heating).

After quenching, HPT was applied to the disks of the alloy 20 mm in diameter and 1.4 mm thick at room temperature (RT) using a unique facility by imposing a pressure of 6 GPa and a strain rate of 0.2 rpm. 

As already known [13,14,15,16], the sample center after HPT processing is normally less worked through and has lower hardness. To exclude this effect, we studied the structure and mechanical properties at a distance of over half the radius from the sample center.

The alloy’s thermal stability in the UFG state was determined by one-hour annealing in a temperature range of 120–270 °C, after which microhardness was characterized and the fine microstructure was analyzed. 

Tensile tests were carried out at room temperature, 190, 240 and 270 °C on an INSTRON 5982 universal testing system (ToolWorks Inc., Norwood, MA, USA) with the Bluehill Universal software (Version 4.08.24426) and computer system for the processing of mechanical characteristics. The strain rates of 10^−2^, 5 × 10^−3^, 10^−3^, 5 × 10^−4^, 10^−4^ and 5 × 10^−5^ s^−1^ were used for the tensile tests. The gauge portion of the tensile test samples was 3.3 mm in length, 1 mm in width and 0.6 mm in thickness.

The mechanical tensile tests were carried out to measure the total elongation to failure and to determine the value of strain-rate sensitivity of flow stress:

$$m=\frac{\partial \ln\left(\sigma \right)}{\partial \ln\left(\dot{\varepsilon }\right)}$$

where σ is the flow stress, $\dot{\varepsilon }$ is the true strain rate.

The microhardness (HV_0.1_) was measured on a Micromet-5101 device (Buehler Ltd., Lake Bluff, IL, USA) with a diamond pyramid indenter, a load of 0.1 N and a dwell time of 15 s. The microhardness values were determined automatically using the OmniMet MHTFS software in the Windows XP operating system.

Microstructural studies were performed using a JEM-2100 transmission electron microscope (JEOL Ltd., Tokyo, Japan) that operated at 200 kV. The disk-shaped samples 3 mm in diameter and 0.1–0.15 mm thick were twin-jet electropolished at a temperature of −25–30 °C and a voltage of 10–12 V and used for analysis by transmission electron microscopy (TEM). Jet polishing of the foils was carried out on a Tenupol-5 automatic electrolytic thinning unit for the TEM samples in a solution of 20% nitric acid (HNO_3_) and 80% methanol grade B (CH_3_OH) at a temperature of (25 + 10) °C and a voltage of 15–20 V.

## 3. Results

### 3.1. Microstructure and Thermal Stability of the UFG 2024 Alloy after Severe Plastic Deformation

**Figure 1:**
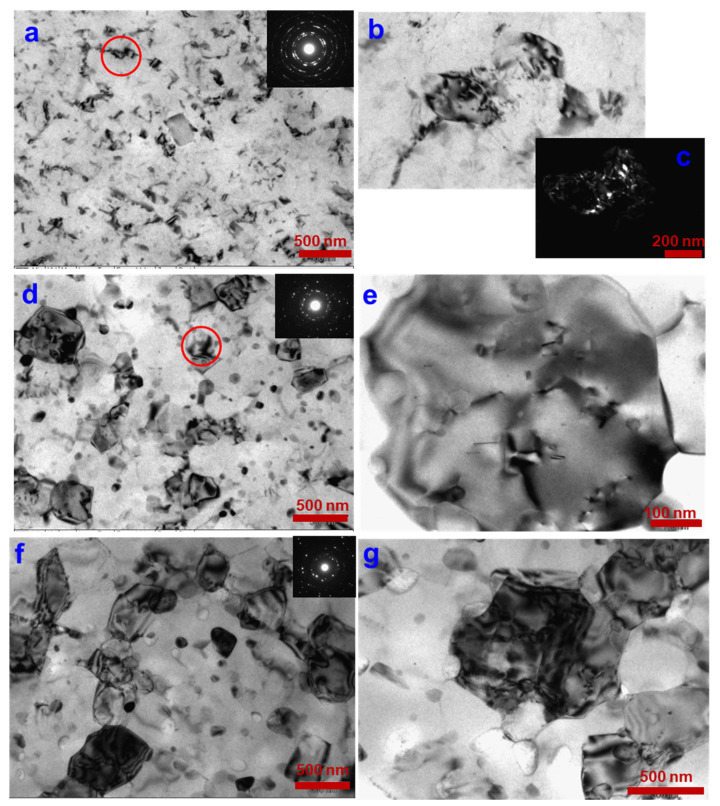
Microstructure after HPT (**a**–**c**); after HPT + annealing at 190 °C (**d**,**e**) and after HPT + annealing at 270 °C (**f**,**g**). The two grains marked by a red circle in Figure 1a are shown in a larger magnification in Figure 1b. The grain marked by a red circle in Figure 1d is shown in a larger magnification in Figure 1e.

As a result of the HPT processing at room temperature, the UFG state of the 2024 alloy was produced, with a mean grain size of 100 ± 7 nm and a grain aspect ratio of 1.2 (Figure 1a). The grain aspect ratio, i.e., the ratio between grain length and grain width, characterizes one of the essential geometrical parameters—grain elongation. In the Al grain interiors, we observed individual globular particles of the strengthening phase with a size up to 5 nm. According to [6,7,8,17], the particles refer to the S(Al_2_CuMg) phase. In addition, authors in [8,17,18,19] noted the formation of Cu segregations at the boundaries of Al grains, which facilitates grain-boundary sliding in the process of superplasticity at lower temperatures [4,20]. The spots are distributed circularly on the diffraction pattern, which is indicative of high-angle misorientations of grain boundaries formed during HPT [21]. Besides the noted reflections, in the electron diffraction patterns additional reflections can be seen (Figure 1a), which belong to the secondary strengthening phase [6,7,8,17].

**Figure 2:**
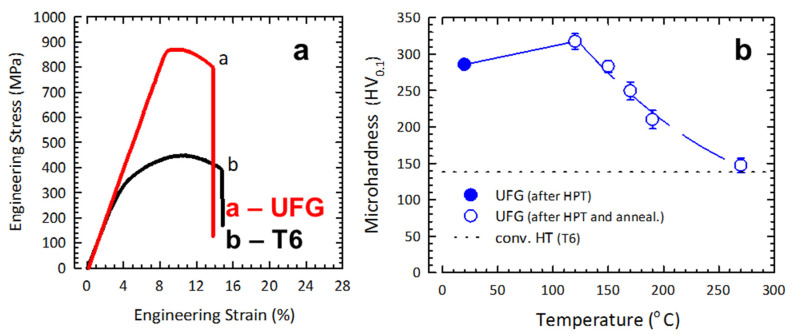
Engineering stress–strain curves for the 2024 alloy in the UFG and coarse-grained states (**a**); dependence of microhardness on temperature (**b**).

The formation of such a state allowed us to achieve, in the 2024 alloy, an offset yield strength (σ_0.2_) of 828 ± 9 MPa, a ultimate tensile strength (σ_UTS_) of 871 ± 6 MPa together with an elongation to failure (δ) of 7.2 ± 0.2%. The strength characteristics of the UFG alloy were two times higher than those after the conventional heat treatment for maximum strength (T6) (Figure 2a). After HPT processing, the microhardness of the UFG 2024 alloy was HV_0.1_ = 286 ± 4, which is two times higher than the alloy’s microhardness after the treatment T6 for maximum strength (HV_0.1_ = 138).

To find the thermal stability of the UFG 2024 alloy, a series of one-hour annealings in a range of 120–270 °C were carried out. The interval of one-hour annealings for finding the alloy’s thermal stability was selected on the basis of the previously published studies, including those on the thermal stability of the 2024 alloy in the UFG state [5,6,7,18]. Figure 2b shows the dependence of microhardness on the temperature of one-hour annealing of the UFG 2024 alloy. Annealing at a temperature of 120 °C leads to an increase in microhardness values to HV_0.1_ = 317 ± 11, which is related to solid solution breakdown induced by the preceding HPT processing and the formation of the strengthening secondary phase. An increase in the temperature of the short-term annealing leads to a gradual decline in microhardness. The material’s microhardness after annealing at 120–190 °C was found to be HV_0.1_ = 300–200. The microhardness after the annealing at 270 °C goes down to the microhardness values of the T6 heat treatment (Figure 2b). In Figure 2b, the straight dotted line denotes the microhardness level after the T6 treatment. The micohardness reduction after one-hour annealing is apparently associated with a decrease in the number of defects in the structure during the processes of recovery and recrystallization of the matrix with a concurrent overaging effect [6].

**Figure 3:**
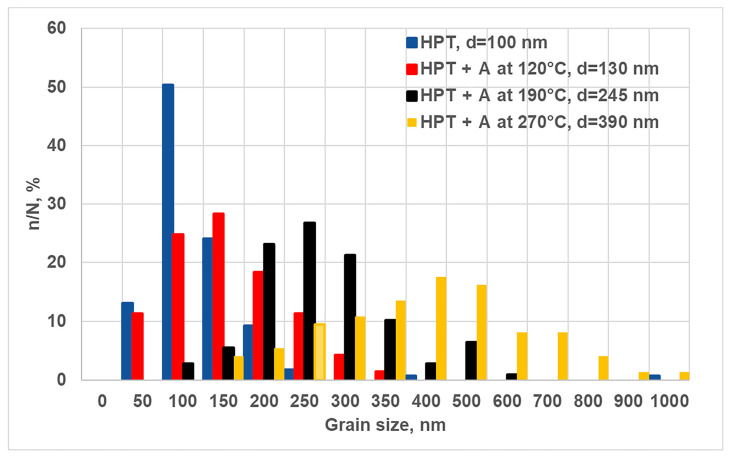
Grain size distributions after HPT and annealing at temperatures of 120, 190 and 270 °C.

The evolution of the microhardness values after annealing is in good agreement with the results from the analysis of microstructural changes. TEM observations show that short-term annealings do not lead to a strong nanostructure degradation, the grain size does not exceed 500 nm, also at a temperature of 270 °C. The mean grain size at a temperature of 120 °C increases to 130 ± 10 nm, and at temperatures of 190 (Figure 1d,e) and 270 °C (Figure 1f,g) it reaches 245 ± 7 and 390 ± 11 nm, respectively. The bar graph showing the grain size distribution in the UFG 2024 alloy after HPT and subsequent one-hour annealing is shown in Figure 3. The grain size peak after the annealing is in a range of 100–150 nm at a temperature of 120 °C; in a range of 200–300 nm at a temperature of 190 °C; in a range of 350–500 nm at a temperature of 270 °C (Figure 3). 

In addition, the TEM analysis revealed the solid solution breakdown with the formation of a secondary strengthening phase both in the Al grain interiors and at grain boundaries after the annealing at 120, 190 and 270 °C (Figure 1). According to [6,7,10], a modification of the strengthening phase S(Al_2_CuMg) is precipitated, and with temperature increasing to 150 °C a modification of the strengthening phase θ(CuAl_2_) is additionally precipitated in a small degree. With the annealing temperature increasing to 270 °C, the process was more intensive than at temperatures of 120 and 190 °C. The size of the globular particles of the secondary strengthening phase was 10–20 nm after annealing at a temperature of 190 °C. The annealing at 270 °C led to the coarsening of particles in ultrafine grains, the particle size was 20–40 nm. With increasing annealing temperature, the density of lattice dislocations decreases, which controls the recovery and recrystallization processes (Figure 1). Thus, a conclusion can be drawn that the 2024 alloy in the UFG state retains its thermal stability up to a temperature of 270 °C.

### 3.2. Mechanical Properties of the UFG Alloy at an Elevated Temperature

The tensile mechanical tests of the 2024 alloy samples in the UFG state were performed in a temperature range of 190–270 °C, where the UFG alloy is expected to retain the high-strength state according to the results from the study of the thermal stability of properties after annealing (Figure 2b). 

**Figure 4:**
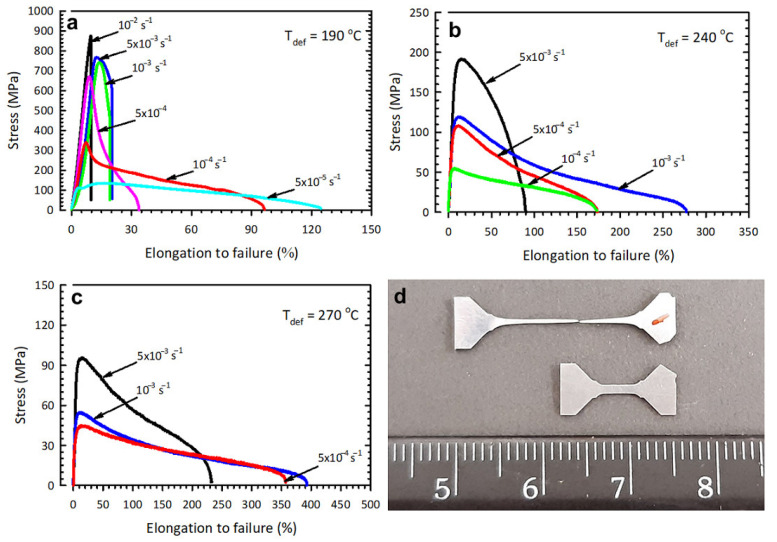
Engineering tensile curves for the UFG Al 2024 alloy subjected to tensile testing at: (**a**) 190, (**b**) 240, (**c**) 270 °C and (**d**) a sample after the test at a temperature of 270 °C and a strain rate of 10^−3^ s^−1^.

The mechanical tests that aimed to find the elongation to failure and the strain-rate sensitivity coefficient (*m*) were conducted at a strain rate range of 10^−2^ to 5 × 10^−5^ s^–1^. Figure 4 shows the tensile curves obtained at deformation temperatures of 190, 240 and 270 °C. 

Interesting results were obtained at a temperature of 190 °C (0.51 T_m_)—the maximum elongations were 130%, but during the transition to the region of low strain rates there was a drastic, 2- to 3-fold, decrease in flow stress, and the strain-rate sensitivity coefficient of flow stress was *m* = 0.51. 

As a result of the tests at a temperature of 240 °C (0.56 T_m_), a maximum elongation of 280% was achieved at a strain rate of 10^−3^ s^−1^, the parameter *m* was 0.32. An increase in the test temperature to 270 °C (0.60 T_m_) led to maximum elongations of 400 and 350% at strain rates of 10^−3^ s^−1^ and 5 × 10^−4^ s^−1^, respectively, and the parameter *m* was 0.33. 

**Figure 5:**
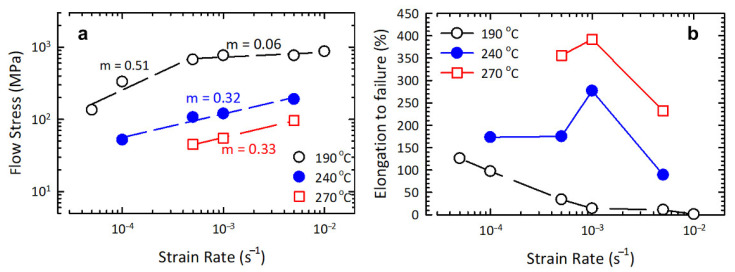
Dependence of flow stress on strain rate (**a**) and elongation on strain rate (**b**), based on the results of static tests at 190, 240 and 270 °C of the UFG Al 2024 alloy.

Figure 5 shows the dependence of elongation to failure and flow stress on strain rate. With increasing deformation temperature, the elongation values grow, at the same time they are displaced towards high strain rates (10^−3^ s^−1^) (Figure 5a). With increasing mechanical test temperature, the flow stresses decline, which is typical of superplastic materials [3,22,23,24,25,26,27]. The test temperatures of 190–270 °C are marked with a reduction in the flow stress value in the strain rate range from 10^−2^ to 5 × 10^−5^ s^–1^. 

Thus, judging by the value of *m* and the attained elongation values, it can be concluded that the UFG 2024 alloy under study exhibits signs of superplastic behavior at lower temperatures.

The microhardness measured after SP deformation is presented in Table 2. Characterization of microhardness in the gauge portion of the samples after mechanical testing shows that microhardness is retained at HV_0.1_ = 160 ± 6 after the superplastic deformation at a temperature of 240 °C, HV_0.1_ = 150 ± 3—at 270 °C. The microhardness of the UFG alloy samples after SP at 270 °C still exceeds that after the T6 treatment for maximum strength (HV_0.1_ = 138) (Table 2).

## 4. Discussion

The formation of the UFG structure in the Al 2024 alloy with a mean grain size of 100 ± 7 nm and an aspect ratio of 1.2 in combination with nanoscale precipitates of the S′(Al_2_CuMg) phase, and segregation of alloying elements in the interiors and boundaries of grains allowed a high strength of 830 MPa to be achieved. The formation of this microstructure in Al alloys enables realizing high elongation values at unusually lower temperatures and high strain rates in the conditions of superplasticity [4,20,27,28]. According to [26,29], fine grains facilitate the grain-boundary sliding and dislocation accommodation; therefore, superplastic behavior can be observed at higher strain rates and lower temperatures. 

The tensile mechanical tests in a temperature range of 190–270 °C have revealed the superplastic behavior of the UFG 2024 alloy where strength is retained but, at the same time, the recovery processes have already started that lead to a slight decline in the material’s number of defects/work hardening, and nanosized globular particles have already formed both in the interiors of the Al grains and at their boundaries, suppressing the recovery and recrystallization processes. 

For the first time, we show that UFG 2024 alloy can demonstrate SP behavior at lower temperatures (0.51 T_m_). The maximum value of elongation 400% was obtained at a temperature of 270 °C, at a strain rate of 10^−3^ s^−1^. The uniform distribution of nanosized precipitates of the S′(Al_2_CuMg) phase, as well as grain boundary segregation [26,29], may play an important role in suppressing dynamic grain growth and ensuring stable flow during SP deformation at this temperature. 

Such a noticeable decrease in the temperature range of superplasticity enables preserving a large part of the unique strength of UFG Al alloys after the realization of SP deformation. In turn, the preservation of the material’s strength should enable avoiding an additional heat treatment or thermomechanical treatment of finished products made of the UFG alloys. It is known that when the conventional conditions of superplasticity are used for the shape forming of products made of Al with a regular fine-grained structure, these types of finishing treatment are used for the restoration or improvement of their strength properties.

## 5. Conclusions

1. The thermal stability range of the UFG 2024 alloy with a grain size of ∼0.1 μm was found to be 120–270 °C, where there was no strong structure degradation, and an enhanced level of microhardness was preserved.
2. Tensile tests were performed at 190–270 °C and strain rates from 10^−2^ to 5 × 10^−5^ s^−1^ to determine ductility and the strain-rate sensitivity of flow stress. Elongations of 280% and *m* = 0.32 were achieved at a temperature of 240 °C and a strain rate of 10^−3^ s^−1^. Elongations of 400% and *m* = 0.33 were achieved for the first time at a lower temperature of 270 °C and a strain rate of 10^−3^ s^−1^. Therefore, it was demonstrated that in the UFG state the 2024 alloy exhibited superplastic behavior at lower temperatures (0.56–0.60) T_m_.
3. The strength of the Al 2024 alloy after low-temperature superplasticity was preserved at a level visibly higher than that after the standard hardening heat treatment T6 of this alloy.

The discovered features of the mechanical behavior of the 2024 alloy with UFG structures may open new opportunities for designing advanced lightweight and high-strength products and structures for the application in both the automotive and aerospace industries.

## Figures and Tables

**Table 1:** Chemical composition of the studied alloy (wt.%).

Cu	Mg	Mn	Si	Fe	Al
4.98 ± 0.01	1.49 ± 0.01	0.73 ± 0.01	0.04 ± 0.01	0.15 ± 0.01	balance

**Table 2:** Microhardness of 2024 Al alloy in different states.

Treatment/ Structural State	T6/Coarse Grained	HPT at RT/UFG	HPT at RT+ SP Deformation at 190 °C/UFG	HPT at RT+ SP Deformation at 240 °C/UFG	HPT at RT+ SP Deformation at 270 °C/UFG
HV_0.1_	138 ± 2	286 ± 4	211 ± 4	160 ± 6	150 ± 3

## Data Availability

The raw and processed data required to reproduce these results are available by reasonable request.
